# First record of *Selenopsab* Logunov & Jäger, 2015 (Araneae, Selenopidae) from China, with first description of the female

**DOI:** 10.3897/BDJ.13.e160680

**Published:** 2025-07-07

**Authors:** Changhao Hu, Yejie Lin, Yang Zhong

**Affiliations:** 1 School of Nuclear Technology and Chemistry & Biology, Hubei University of Science and Technology, Xianning, Hubei, China School of Nuclear Technology and Chemistry & Biology, Hubei University of Science and Technology Xianning, Hubei China; 2 Hubei Broad Nature Technology Service Co., Ltd., Wuhan, China Hubei Broad Nature Technology Service Co., Ltd. Wuhan China; 3 Department of Life Sciences, Imperial College London, London SW7 2AZ, United Kingdom Department of Life Sciences, Imperial College London London SW7 2AZ United Kingdom

**Keywords:** new record, morphology, biodiversity, taxonomy

## Abstract

**Background:**

*Selenopsab* Logunov & Jäger, 2015 was originally described, based on a male specimen collected from Vietnam, with the female remaining unknown.

**New information:**

Based on specimens collected from Shenzhen City, Guangdong Province, the female of *Selenopsab* Logunov & Jäger, 2015 is described for the first time. This species is newly recorded from China.

## Introduction

*Selenops* Latreille, 1819 is the largest genus in the family Selenopidae Simon, 1897, comprising 132 extant species distributed across Africa, Asia, Europe, North America and South America (World Spider Catalog 2025). *Selenops* spiders are nocturnal, possess a flattened body and typically inhabit crevices in tree trunks, rocks and the walls of houses (Yin et al. 2012, Zhang and Wang 2017). In China, only four *Selenops* species have been recorded to date: *S.bursarius* Karsch, 1879, *S.crewsae* Lin & Li, 2021, *S.ollarius* Zhu, Sha & Chen, 1990 and *S.radiatus* Latreille, 1819 (Song et al. 1999, Lin et al. 2021).

*Selenopsab* Logunov & Jäger, 2015 was originally described, based on a male specimen from Vietnam (Logunov and Jäger 2015) and the female of this species has remained unknown. The current paper provides the first description of the female of *S.ab*, based on specimens collected from Shenzhen City, Guangdong Province, China. This also represents the first record of this species in China.

## Materials and methods

All specimens were preserved in 80% ethanol. The spermathecae were cleared in trypsin enzyme solution to dissolve non-chitinous tissues. Specimens were examined under a Leica M205C stereomicroscope. Photomicrographs were taken with an Olympus C7070 zoom digital camera (7.1 megapixels). Laboratory habitus photographs were taken with a Sony A7RIV digital camera equipped with a Sony FE 90 mm Goss lens. Photos were stacked with Helicon Focus® (Version 7.6.1) or Zerene Stacker®(Version 1.04) and processed in Adobe Photoshop CC2022. The map was created with ArcGis v. 10.8.1 (Esri 2020).

All measurements are in millimetres (mm) and were obtained with an Olympus SZX16 stereomicroscope with a Zongyuan CCD industrial camera. All measurements of body lengths do not include the chelicerae. Eye sizes are measured as the maximum diameter from either the dorsal or frontal view. Leg measurements are given as follows: total length (femur, patella + tibia, metatarsus, tarsus). The type materials are deposited in the Institute of Zoology, Chinese Academy of Sciences in Beijing (IZCAS).

The terminologies were modified from Crews (2011) and Zamani and Crews (2019). Abbreviations: **ALE** = anterior lateral eye; **AME** = anterior median eye; **C** = conductor; **CD** = copulatory duct; **CO** = copulatory opening; **dRTA** = dorsal branch of retrolateral tibial apophysis; **E** = embolus; **EP** = epigynal pocket; **FD** = fertilisation duct; **MA** = median apophysis; **PF** = posterodorsal fold; **PLE** = posterior lateral eye; **PME** = posterior median eye; **PS** = primary spermatheca; **SH** = spermathecal head; **SS** = secondary spermatheca; **vRTA** = ventral branch of retrolateral tibial apophysis; **I, II, III, IV** = legs I to IV.

## Taxon treatments

### Selenops ab

Logunov & Jäger, 2015

357E186D-5A57-5C26-A463-443D901FB00E

*Selenopsab Logunov and Jäger 2015*: 348, figs. 12‒16 (male).

#### Materials

- **Type status:** Other material. **Occurrence:** recordedBy: Qianle Lu; individualCount: 2; sex: 1 male, 1 female; lifeStage: adult; **Location:** continent: Asia; country: China; countryCode: CN; stateProvince: Guangdong Province; county: Shenzhen City, Luohu District; verbatimLocality: Wutong Mountain; verbatimElevation: 490 m (obtained from Google Earth); verbatimLatitude: 22°34’54” N (obtained from Google Earth); verbatimLongitude: 114°12’19” E (obtained from Google Earth); **Event:** year: 2019; month: 3; day: 15

#### Description

**Female**: Total length 6.28; carapace length 2.50, carapace width 2.57; opisthosoma length 3.98, opisthosoma width 2.61. Eye measurements: AME 0.12; ALE 0.08; PME 0.13; PLE 0.20; AME‒AME 0.17; AME‒ALE 0.32; PME‒PME 0.56; PME‒PLE 0.27; AME‒PME 0.09; ALE‒PLE 0.14. Measurements of legs: I 6.01 (2.05, 2.36, 1.04, 0.56); II 7.30 (2.59, 2.73, 1.45, 0.53); III 7.73 (2.96, 2.56, 1.58, 0.63); IV 6.37 (2.54, 2.03, 1.35, 0.45). Leg formula: III-II-IV-I. Promargin and retromargin of chelicerae with two teeth.

**Figure 1.**
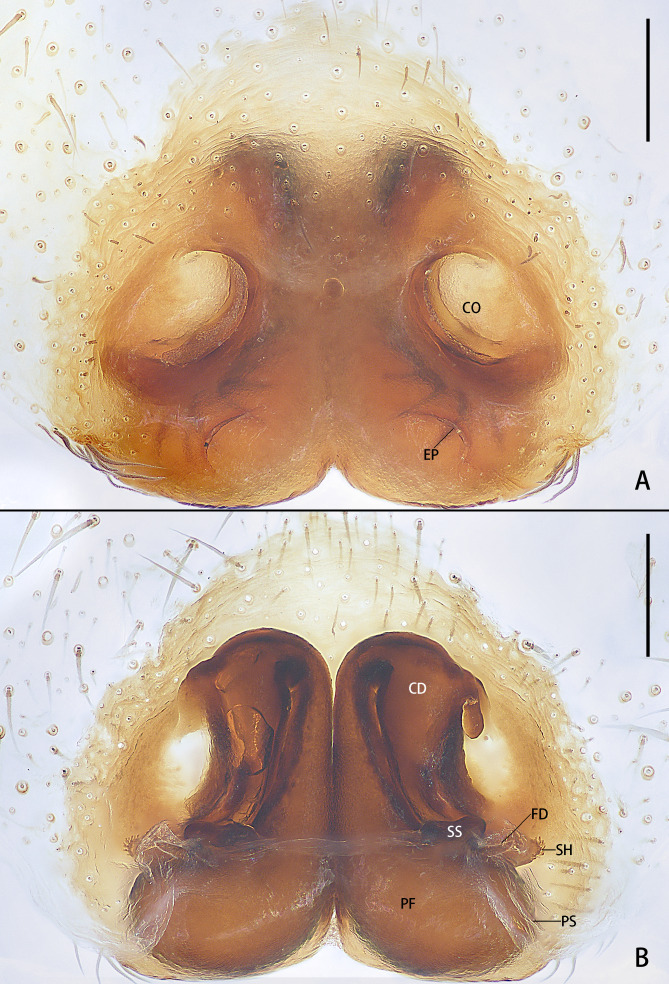
Epigyne of *Selenopsab* Logunov & Jäger, 2015. **A** Ventral view; **B** Dorsal view. Abbreviations: **CD** = copulatory ducts; **CO** = copulatory opening; **EP** = epigynal pocket; **FD** = fertilisation duct; **PF** = posterodorsal fold; **PS** = primary spermatheca; **SH** = spermathecal head; **SS** = secondary spermatheca. Scale bars: 0.2 mm.

**Epigyne** (Fig. 1): Epigynal field almost inverted heart-shaped. Copulatory openings (CO) oval, laterally located. Posterior part of epigyne with pair of epigynal pockets (EP). Copulatory ducts (CD) thick, with thin turning part. Primary spermathecae (PS) water-drop-shaped, laterally with spermathecal heads (SH) and heavily sclerotised secondary spermathecae (SS); secondary spermathecae (SS) almost as small as spermathecal heads (SH). Fertilisation ducts (FD) straight, shorter than spermathecal heads (SH), located between spermathecal heads (SH) and secondary spermathecae (SS). Posterodorsal folds (PF) membranous, trapezoidal.

**Colouration** (Fig. 3A): Carapace almost rounded, light brown, with dark brown fovea and thick hairs. Palps and legs yellow, with brown markings. Opisthosoma yellow, with brown spots, posteriorly with two brown sub-triangular markings.

**Figure 2.**
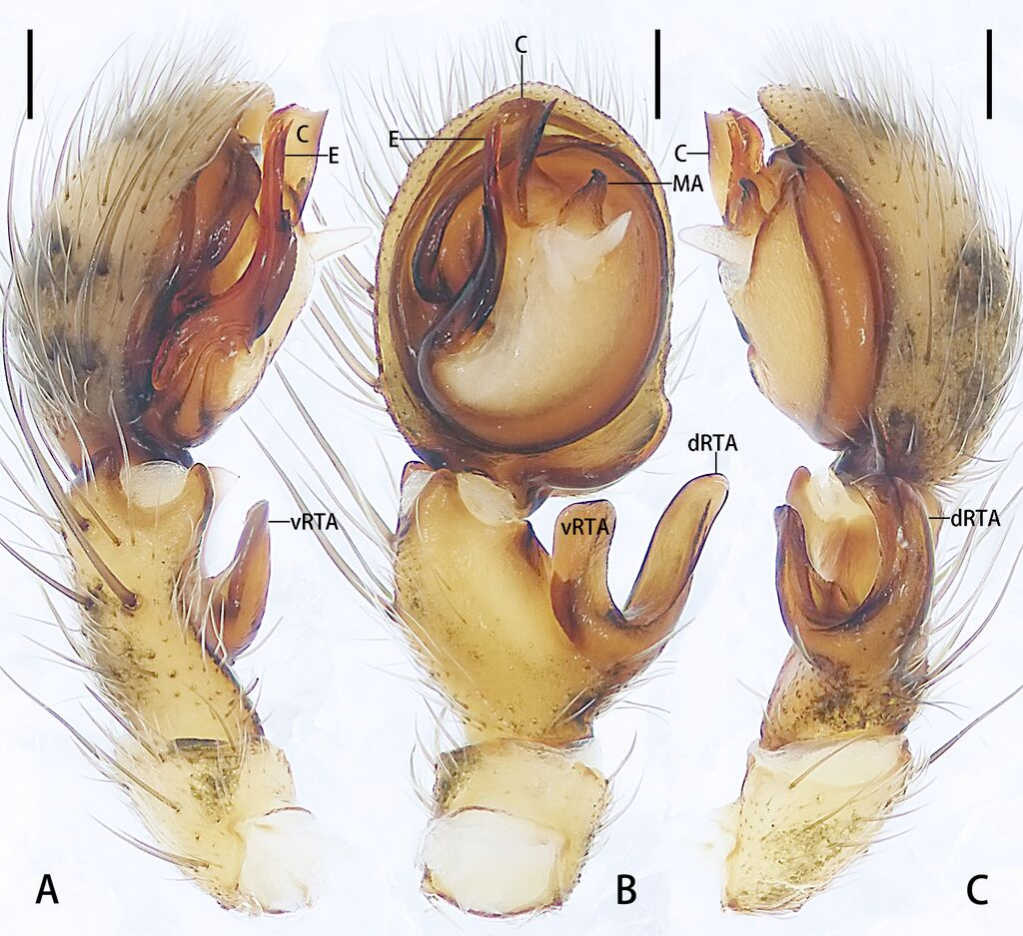
Left male palp of *Selenopsab* Logunov & Jäger, 2015. **A** Prolateral view; **B** Ventral view; **C** Retrolateral view. Abbreviations: **C** = conductor; **dRTA** = dorsal branch of retrolateral tibial apophysis; **E** = embolus; **MA** = median apophysis; **vRTA** = ventral branch of retrolateral tibial apophysis. Scale bars: 0.2 mm.

**Figure 3.**
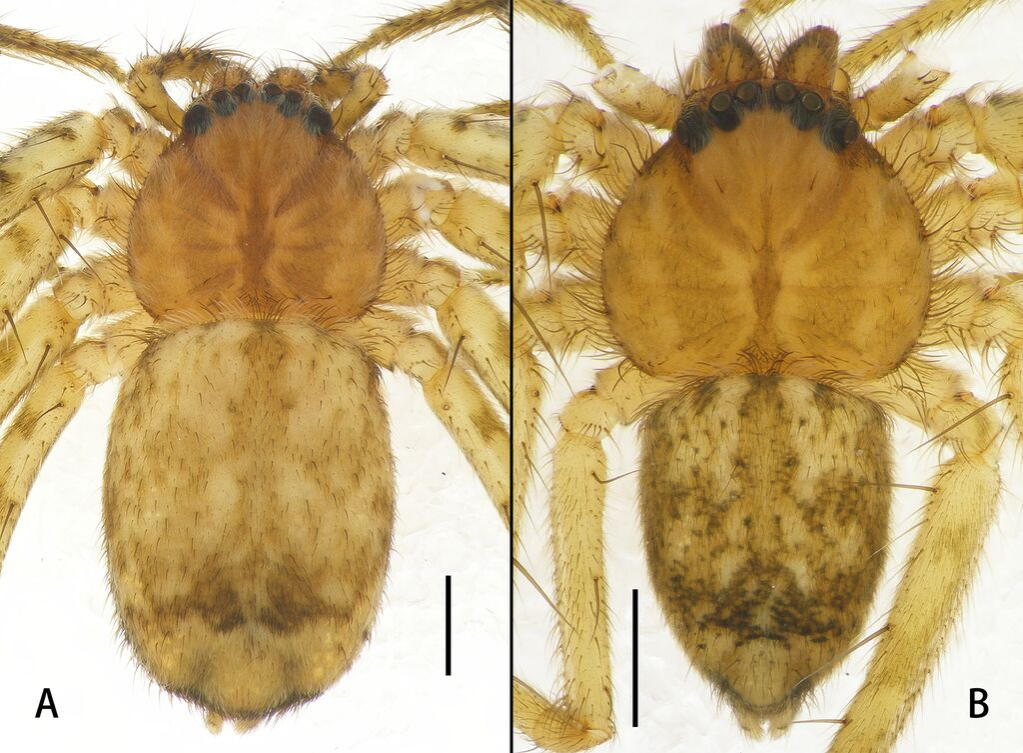
Habitus of *Selenopsab* Logunov & Jäger, 2015, dorsal view. **A** Female; **B** Male. Scale bars: 1 mm.

**Male** (Figs 2, 3B): See Logunov and Jäger (2015).

#### Diagnosis

The female of *Selenopsab* Logunov & Jäger, 2015 is similar to those of *S.lumbo* Corronca, 2001 (cf. Fig. 1 and figs. 51, 52 in Corronca (2002)) in having two distant copulatory openings (CO) laterally, but can be distinguished from it by: 1. epigynal pockets (EP) posteriorly located (vs. medially located); 2. turning part of copulatory ducts (CD) thin, almost 1/3 as wide as the rest (vs. thick, almost as wide as copulatory ducts (CD)); 3. primary spermathecae (PS) water-drop-shaped (vs. double spherical); and 4. fertilisation ducts (FD) short and straight (vs. long and curved). For the male, see Logunov and Jäger (2015).

#### Distribution

**Figure 4.**
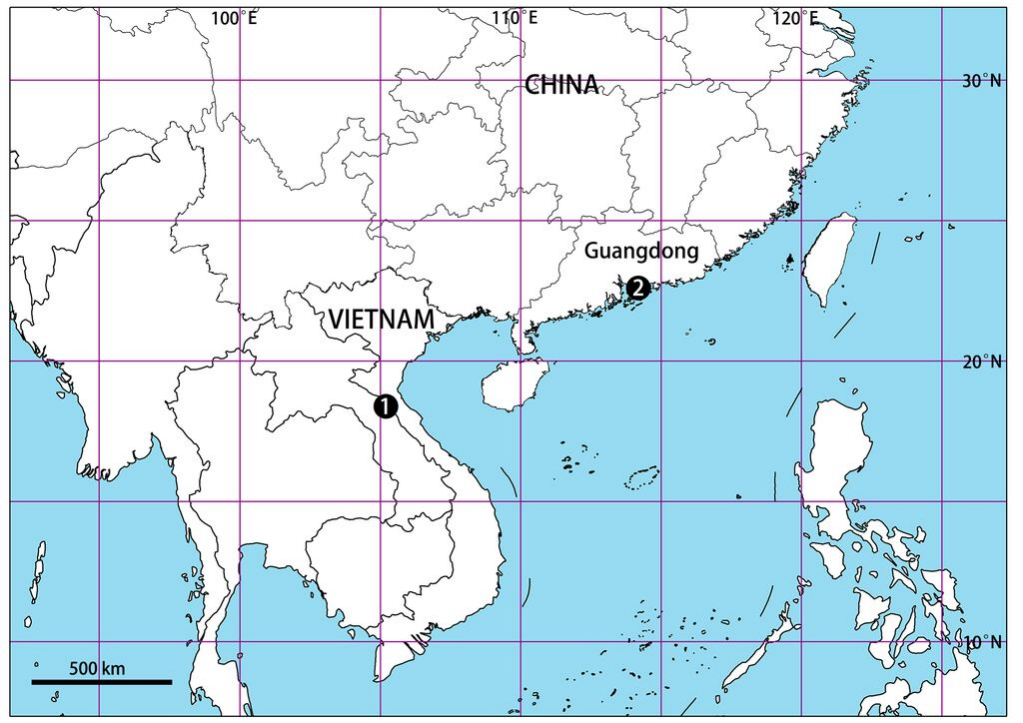
Distribution map of *Selenopsab* Logunov & Jäger, 2015. **1** Type locality; **2** New record from China.

China (Guangdong; new record), Vietnam (Fig. 4).

#### Biology

**Figure 5.**
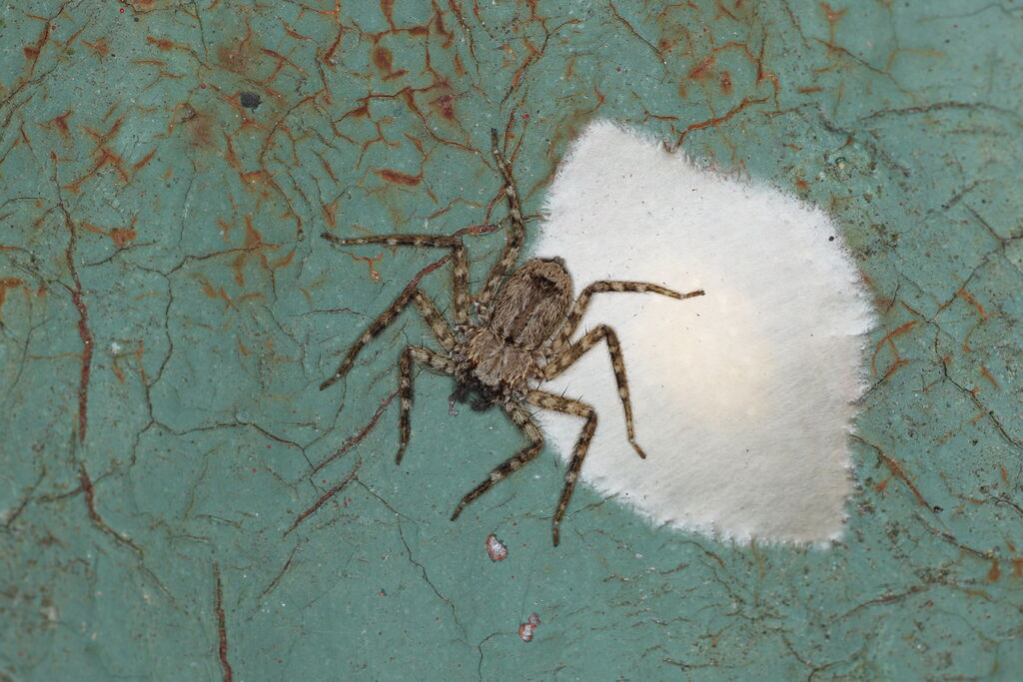
Living female of *Selenopsab* Logunov & Jäger, 2015 guarding an egg sac (Photo by Qianle Lu).

This species was found in crevices of tree trunks and on railings in Wutong Mountain, Shenzhen (Fig. 5).

## Supplementary Material

XML Treatment for Selenops ab
